# Supplementary material for: Selenium Modulates the Allergic Response to Whey Protein in a Mouse Model for Cow’s Milk Allergy
Source: Nutrients. 2021 Jul 22;13(8):2479. doi: 10.3390/nu13082479 (PMC8400770; doi:10.3390/nu13082479)
Supplement: Supplementary file 1 [file nutrients-13-02479-s001.zip › 2021-07-08 nutrients-1291258 - 4. Supplementary Materials combined.pdf]

Figure S1.

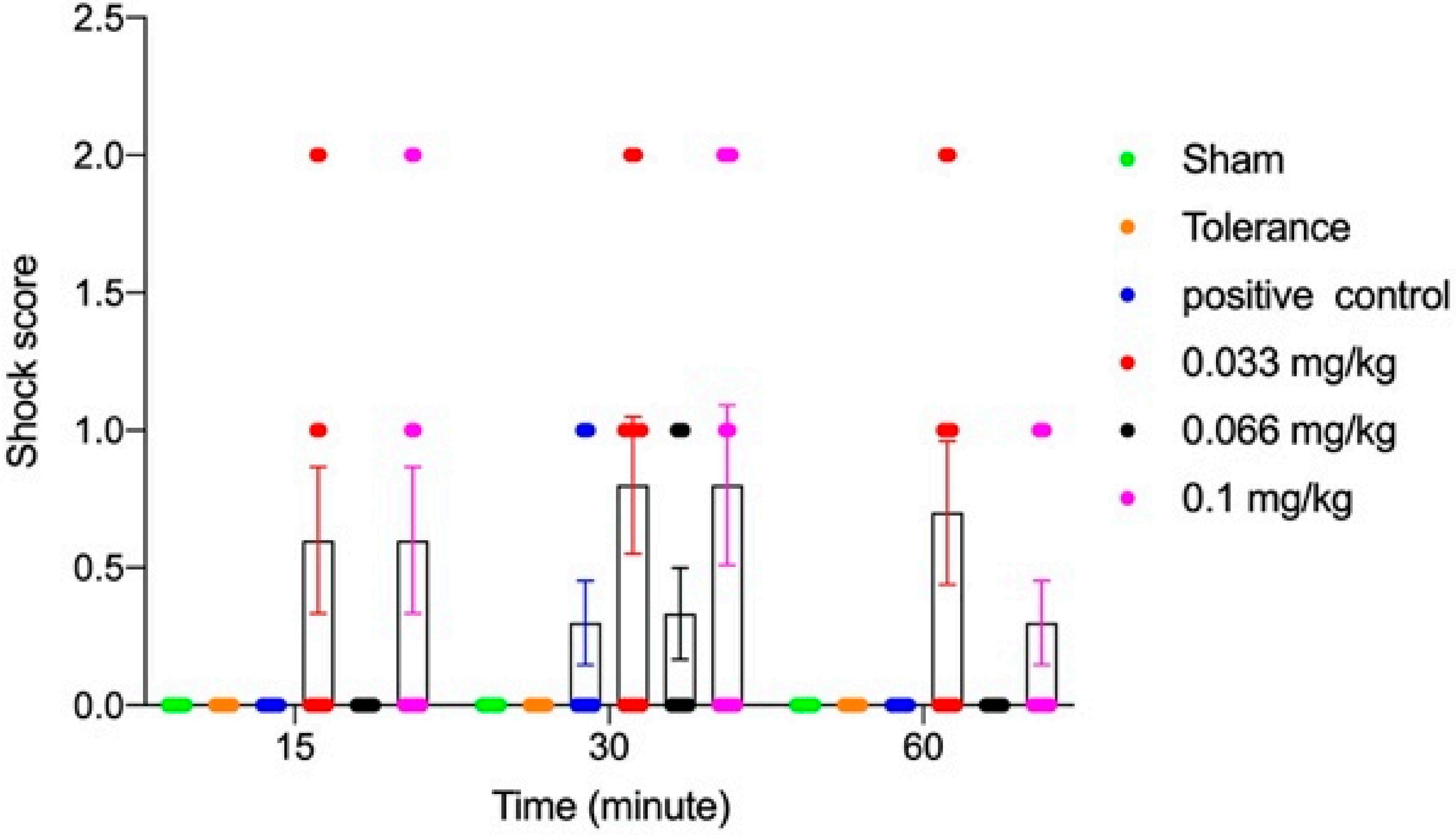

Figure S1. The acute allergic anaphylactic shock score of mice fed the control or the Se diets. Anaphylactic shock score was monitored 15, 30, and 60 minutes after i.d. challenge. Data are presented as mean ± SEM of n = 4-10/group including individual data points. Differences are analyzed with a two-way analysis of variance (ANOVA) followed by a Gesser-Greenhouse test.

Figure S2.

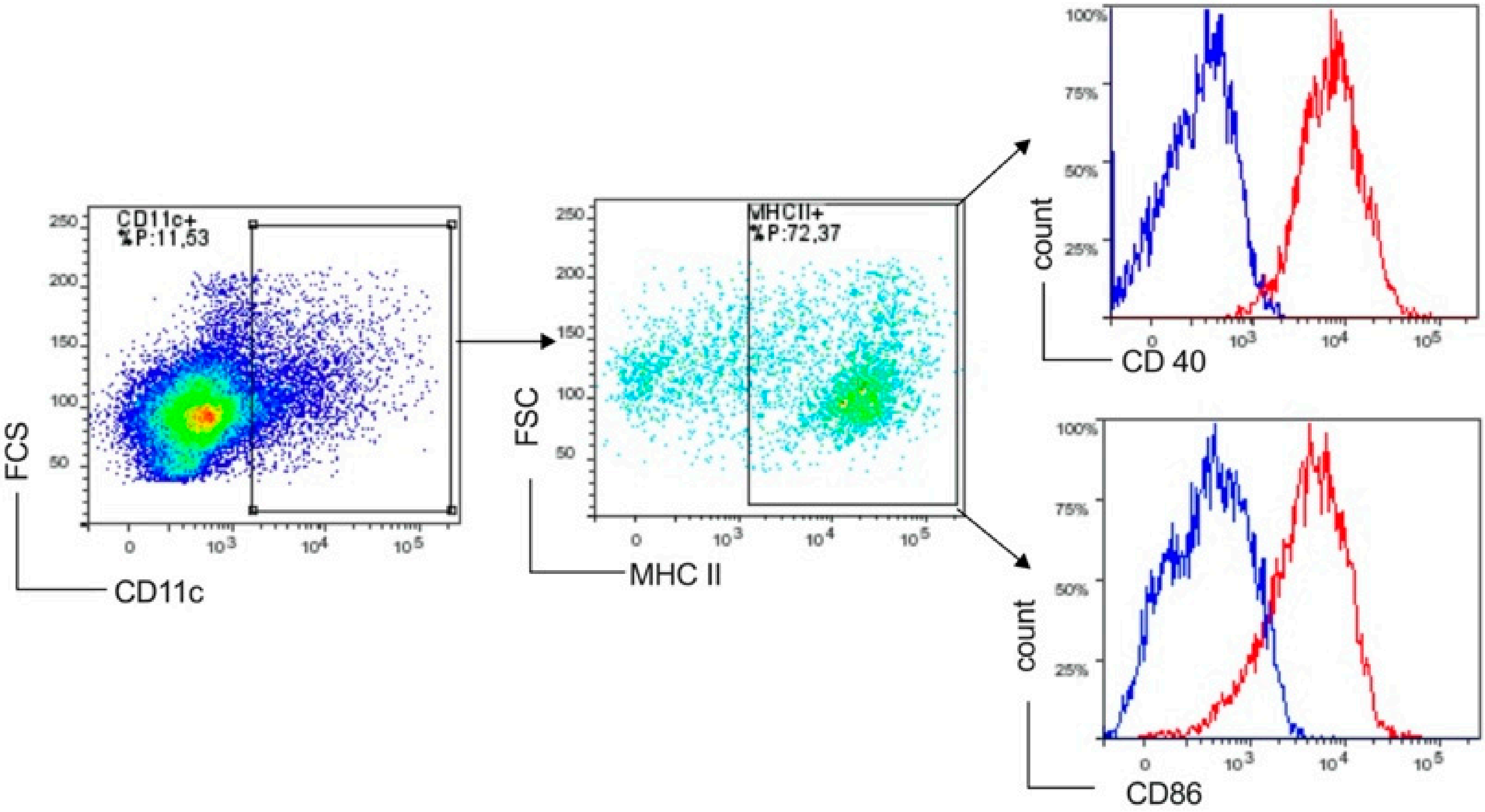

Figure S2. Gating strategy of splenic dendritic cells (DC). Single cells were collected and analyzed by flow cytometry. Cells were stained for viability using FVD (APC-Cyc7), followed by extracellular staining of CD11c-PerCp-Cy5.5, MHCII-APC, CD40-FITC and CD86-PE-cy7. Flow logic was used to analyze the data. In the gating strategy, single cells were selected based on FSC-A and FSC-H, prior to selection of live cells. Out the gate of live cells, CD11c + MHC-II+ cells were selected by FMO control. Next, CD40+ and CD86+ were selected out the gate of CD11c + MHC-II+ using FMO controls.

Figure S3

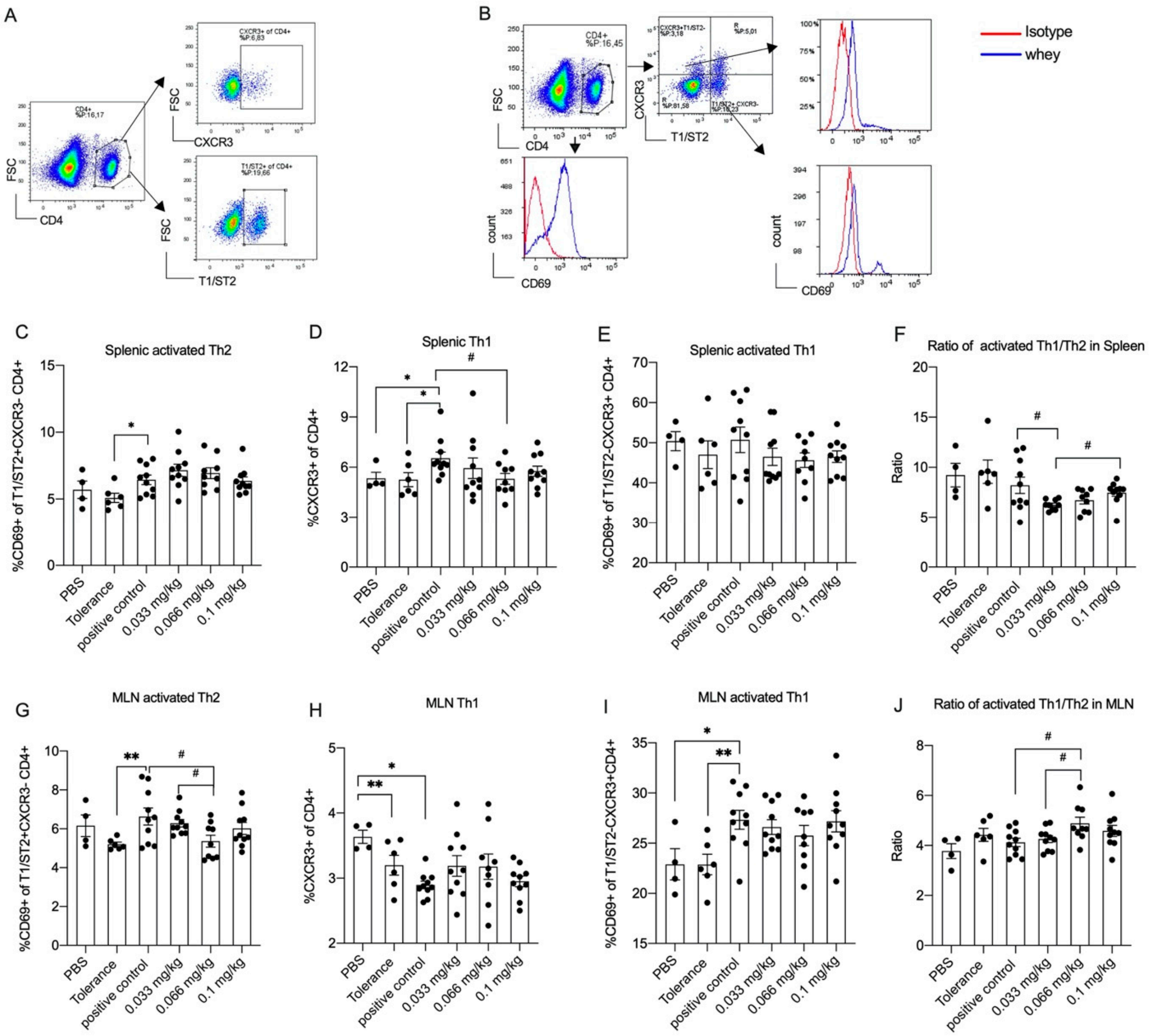

A
B
C
Splenic activated Th2
D
Splenic Th1
E
Splenic activated Th1
F
Ratio of activated Th1/Th2 in Spleen
G
MLN activated Th2
H
MLN Th1
I
MLN activated Th1
J
Ratio of activated Th1/Th2 in MLN
Isotype
whey
PBS
Tolerance
positive control
0.033 mg/kg
0.066 mg/kg
0.1 mg/kg

Figure S3. Flow cytometric analysis of T helper in Spleen and MLN at day 36. Gating strategy of Th1 and Th2 cells in the spleen and MLN (A, B). Single cells were collected and analyzed by flow cytometry. The cells were stained for viability using FVD (APC-Cyc7), followed by exocellular staining of CD4, CD69, CXCR3 and T1/ST2. Flow logic was used to analyze the data. In the gating strategy, the single cell was first selected based on FSC-A and FSC-H, then live cells were selected. Out the gate of live cell, the CD4+ cell was selected by FMO control. Next, CXCR3 or T1/ST2 were selected out the gate of the CD4+ using FMO controls. For activated Th1, CD69+ were selected out of the gate of CXCR3+ T1/ST2- of CD4+. For activated Th2, CD69+ were selected out of the gate of CXCR3- T1/ST2+of CD4+. Th1 cells were defined as CXCR3+ of CD4+ cells. Activation-status was determined by CD69 positivity of the cells. Percentages of splenic (C) activated Th2-cells; (D) Th1-cells; (E) activated Th1-cells and (F) Ratio of activated Th1/Th2 cells. Percentages of MLN (G) activated Th2-cells; (H) Th1-cells; (I) activated Th1-cells and (J) activatedTh1/Th2 ratio. Data are presented as mean ± SEM of PBS group n=4, tolerance group n=6, positive control group or low and high Se intervention groups n=10/group, medium Se intervention group n=9 including individual data points; Significant differences between PBS, tolerance and whey-sensitized mice are indicated by *P < 0.05 and **P <0 .01. Differences between whey-sensitized mice fed with the control diet and those fed with the Se diets are indicated by # P <0 .05 and ## P < 0.01. Differences are analyzed with a one-way analysis of variance (ANOVA) followed by a Kruskal Wallis' non-parametric test.

Figure S4

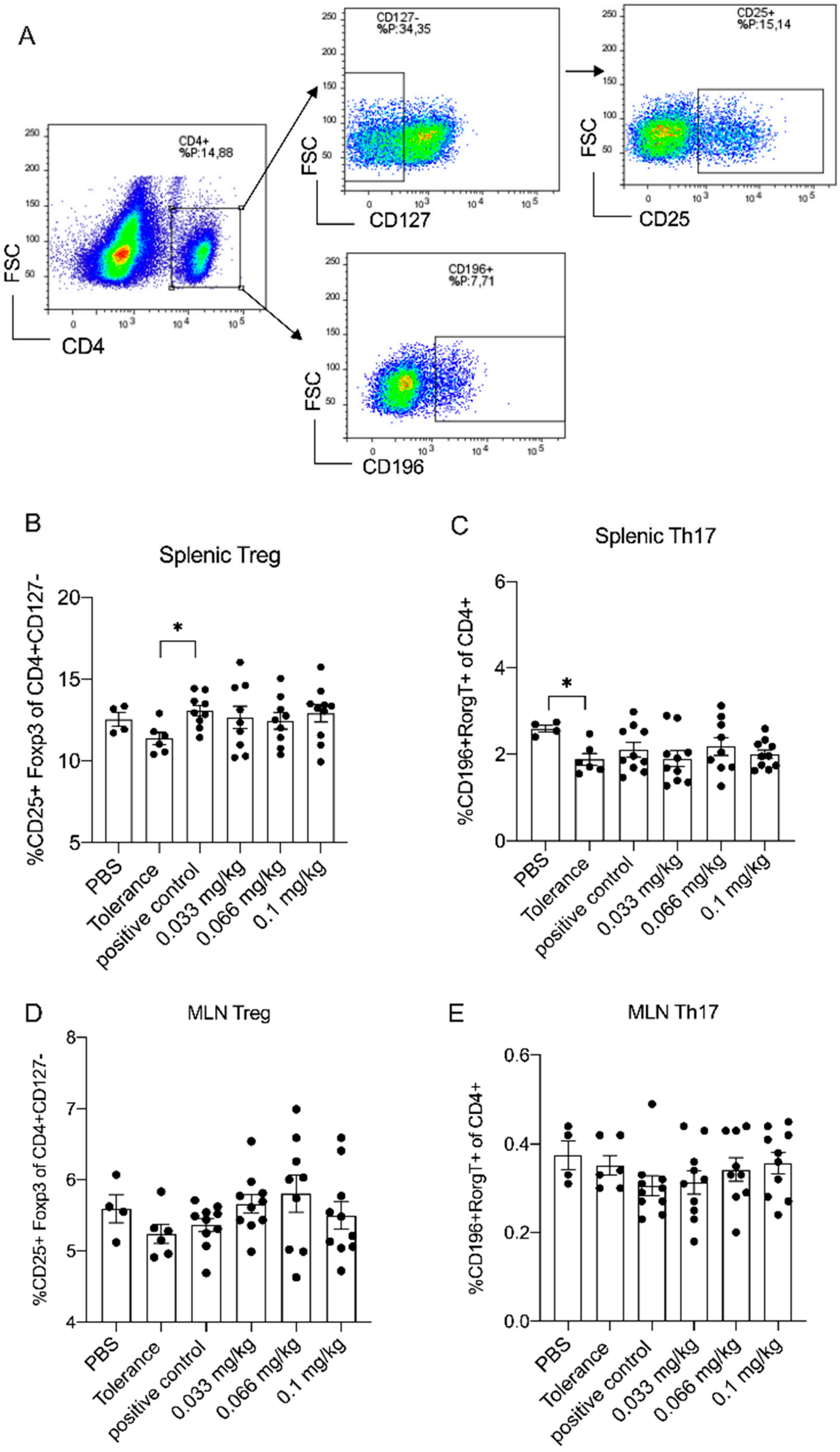

A
CD4+
%P:14,88
FSC
CD4
CD127-
%P:34,35
FSC
CD127
CD25+
%P:15,14
FSC
CD25
CD196+
%P:7,71
FSC
CD196
B
Splenic Treg
%CD25+ Foxp3 of CD4+CD127-
PBS
Tolerance
positive control
0.033 mg/kg
0.066 mg/kg
0.1 mg/kg
C
Splenic Th17
%CD196+RorgT+ of CD4+
D
MLN Treg
%CD25+ Foxp3 of CD4+CD127-
E
MLN Th17
%CD196+RorgT+ of CD4+

Figure S4. Flow cytometric analysis of regulatory T cell (Tregs) populations and T helper (Th) 17 populations in the spleen and MLN at day 36. (A) Gating strategy. Single cells were collected and analyzed by flow cytometry. The cells were stained for viability using FVD (APC-Cyc7), followed by extracellular staining of CD4, CD127, CD25 and CD196, and intracellular staining of Foxp3 and RorgT. Flow logic was used to analyze the data. In gate strategy, the single cell was first selected based on FSC-A and FSC-H, then live cell was selected. Out the gate of live cell, the CD4+ cell was selected by FMO control. CD127- were selected by FMO control out the gate of CD4+. Next, CD25+ Foxp3+ were selected by FMO control out the gate of CD127- CD4+ or CD196+ RorgT were selected out the gate of the CD4+ using FMO controls. (B) Percentage of splenic Treg (CD25+Foxp3+ of CD127-CD4+ cells); (C) Percentage of splenic Th17-cells (CD196+RorgT of CD4+ cells). (D) Percentage of MLN Treg (CD25+Foxp3+of CD127-CD4+ cells); (E) Percentage of MLN Th17-cells (CD196+RorgT of CD4+ cells). Significant differences between PBS, tolerance and whey-sensitized mice are indicated by *$P < .05$ and **$P < .01$. Differences between whey-sensitized mice fed with the control diet and those fed with the Se diet are indicated by # $P < .05$. Differences are analyzed with a one-way analysis of variance (ANOVA) followed by a Kruskal Wallis' non-parametric test.

Figure S5

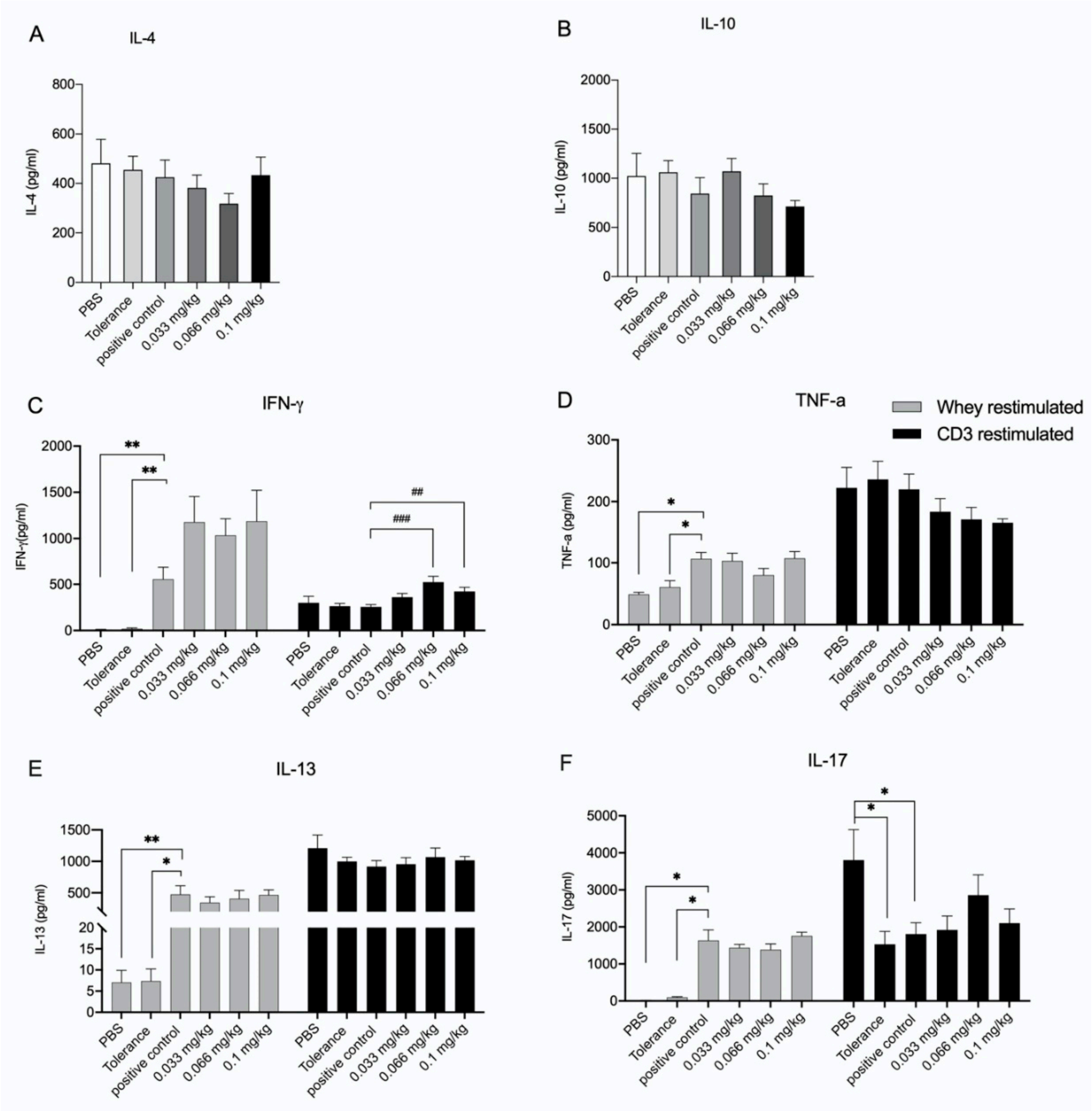

Figure S5. Cytokines concentrations were measured in supernatant of *ex vivo* stimulated splenocytes with whey or antiCD3. (A) IL-4 production in antiCD3 restimulated splenocytes. (B) IL-10 production in antiCD3 restimulated splenocytes. (C) IFN-γ production in whey or antiCD3 restimulated splenocytes. (D) TNF-α in whey or antiCD3 restimulated splenocytes. (E) IL-13 in whey or antiCD3 restimulated splenocytes. (F) IL-17 in whey or antiCD3 restimulated splenocytes. Data are presented as mean ± SEM. Significant differences between PBS, tolerance and whey-sensitized mice are indicated by *$P < .05$ and **$P < .01$. Differences between whey-sensitized mice fed with the control diet and those fed with the Se diet are indicated by # $P < .05$ and ##$P < .01$. Differences are analyzed with a one-way analysis of variance (ANOVA) followed by a Kruskal Wallis' non-parametric test.
