# Supplementary material for: Selenium Modulates the Allergic Response to Whey Protein in a Mouse Model for Cow’s Milk Allergy
Source: Nutrients. 2021 Jul 22;13(8):2479. doi: 10.3390/nu13082479 (PMC8400770; doi:10.3390/nu13082479)
Supplement: Supplementary file 1 [file nutrients-13-02479-s001.zip › 2021-07-08 nutrients-1291258 - 7. Supplementary materials accompanying text.pdf]

1. Dietary Se modestly affects T cell differentiation

Se-supplementation did not affect Th2 activation status (Figure S3 C). The percentages of splenic Th1 cells were significantly higher in the positive control group when compared to the PBS group and tolerance group (Figure S3 D), and it was significantly lower in medium Se intervention group compared to the positive control group (Figure S3 D). However, no differences in percentages of CD69+ activated Th1 were detected (Figure S3 E). Th1/Th2 ratios did not appear to be significantly affected as no significant differences were observed when the PBS group, the tolerance group and the positive control group were compared to each other. However, compared to positive control group, the Th1/Th2 ratio was significantly decreased in the low Se group, and it was significantly increased in high Se group compared to the low Se group (Figure S3 F). In contrast to what was observed in the spleen, the percentage of Th1 cells was significantly lower in the MLNs of mice in the positive control group compared to the PBS and the tolerance group (Figure S3 H). Se supplementation did not appear to affect the percentage of Th1 cells (Figure S3 F). However, percentages of CD69+ activated Th1 cells were higher in in the positive control group when compared to the PBS group and the tolerance group (Figure S3 I). No significant differences in activated Th2 cells (Figures S3 G) or Th1/Th2 ratios (Figures S3 J) could be detected when the positive control group was compared to the PBS group, although these parameters were significantly different when the medium Se group was compared to the positive control group.

In the spleen, the percentages of Tregs and Th17 cells were not significantly affected by sensitization as no differences between the negative and positive control group were observed although these percentages of the T-cell subsets were significantly higher in the positive control animals when compared to the tolerance group (Figures S4 B, C), Se-supplementation did not affect Treg populations, but when compared to the positive control group the percentage of Th17 cells was significantly lower in the medium Se-group (Figures S4 B, C). In the MLN, no significant differences in percentages of Treg or Th17 cell were observed (Figures S4 D, E).
